# A diagnostic challenge for schistosomiasis japonica in China: consequences on praziquantel-based morbidity control

**DOI:** 10.1186/1756-3305-4-194

**Published:** 2011-10-07

**Authors:** Yi-biao Zhou, Hui-min Zheng, Qing-wu Jiang

**Affiliations:** 1 Department of Epidemiology, School of Public Health, Fudan University, 138 Yi Xue Yuan Road, Shanghai 200032, China; 2 Key Laboratory of Public Health Safety, Ministry of Education (Fudan University), 138 Yi Xue Yuan Road, Shanghai 200032, China

**Keywords:** Schistosomiasis, *Schistosoma japonicum*, Parasitological examination, Immunodiagnosis, Chemotherapy

## Abstract

Worldwide schistosomiasis continues to be a serious public health problem. Over the past five decades, China has made remarkable progress in reducing Schistosoma japonicum infections in humans to a relatively low level. Endemic regions are currently circumscribed in certain core areas where re-infection and repeated chemotherapy are frequent. At present, selective chemotherapy with praziquantel is one of the main strategies in China's National Schistosomiasis Control Program, and thus diagnosis of infected individuals is a key step for such control. In this paper we review the current status of our knowledge about diagnostic tools for schistosomiasis japonica. A simple, affordable, sensitive, and specific assay for field diagnosis of schistosomiasis japonica is not yet available, and this poses great barriers towards full control of schistosomiasis. Hence, a search for a diagnostic approach, which delivers these characteristics, is essential and should be given high priority.

## Background

Schistosomiasis or 'bilharzia' continues to be a serious public health problem worldwide. The main disease-causing species are *Schistosoma japonicum, S. mansoni*, and *S. haematobium*, which affect more than 200 million people in approximately 70 countries, resulting in a loss of 1.53 million disability-adjusted life years [1,2]. Schistosomiasis japonica is one of the major tropical diseases in China, with a documented history of > 2,100 years [3]. In the mid 1950s at the beginning of the National Control Program, schistosomiasis was endemic in 4,078 townships, belonging to 433 counties/cities of 12 provinces and the estimates of the maximum number of people infected ranged between 10.5 and 11.8 million [4,5]. From the mid-1950s to 1980s, comprehensive control measures were carried out with an emphasis on the control of the intermediate host i.e. the snail, by means of environmental management, which targeted the interruption of transmission. Great success was accomplished in four out of twelve endemic provinces and in two-thirds of the endemic counties/cities. Endemic regions were then circumscribed in certain core areas, such as the middle and low reaches of the Yangtze River as well as endemic hilly and mountainous regions in Sichuan and Yunnan provinces, where achieving sustainable control is very difficult and the endemic levels fluctuated in the 1980s [6]. Following the discovery of praziquantel, the global strategy of schistosomiasis control shifted from transmission control to morbidity control [7]. In China the praziquantel-based morbidity control strategy has been practiced on a large scale in endemic regions since the 1990s, especially during the 10-year World Bank Loan Project (WBLP), which ran from 1992 to 2001 [8]. The consequence of such an effective control program based on mass or selective chemotherapy was a significant reduction in prevalence and infection intensities with *Schistosoma japonicum *in humans [8]. At present, selective chemotherapy with praziquantel is one of the main strategies for control of schistosomiasis in the National Schistosomiasis Control Program, and thus, diagnosis is a key factor for schistosomiasis control [9]. Determination of target populations for treatment in the endemic areas, assessment of morbidity, and the evaluation of control programs all build on the diagnostic tests.

In general, *S. japonicum *infections can be diagnosed by three different approaches: a) to detect schistosome eggs in fecal samples by direct parasitological methods or eggs in tissue biopsies by histological methods; b) to test immunological responses to certain schistosome antigens and the levels of parasite-derived antigens in blood and urine; and c) to measure pathological morbidity associated with schistosome infection by clinical, subclinical, and biochemical markers. There are other methods such as questionnaires, but their specificity is questionable. Although the histological method is a sensitive and specific clinical diagnostic method, it is neither simple nor convenient for population-based surveys. There are many methods for measuring the morbidity associated with *S. japonica*, e.g. clinical, ultrasonography, but they lack specificity in some areas such as China.

### Direct parasitological examinations

Detection of parasite eggs in stool samples is a traditional and still widely employed method for diagnosing schistosome infections. There are many variations of direct parasitological examinations, but the Kato-Katz thick smear (KK) [10] and the miracidium hatching test (MHT) [11] are the two most widely used in the field in China.

The KK method is the most extensively used method for diagnosing *S. japonica *in field surveys because it is quantitative, relatively inexpensive, and simple. However, it has become relatively insensitive following widespread chemotherapy, which results in generally low worm burdens [12]. The specificity and positive predictive value (PPV) of the KK method are good irrespective of the reference gold standards and the infection rates in humans, but the sensitivities of the KK method vary from 40% to 100%, and the negative predictive values (NPV) of the KK method range from 52.5% to 100% (Table 1) [13-20]. Table 1[13-20] shows that the last two parameters (sensitivity and NPV) of the KK method are highly dependent on the prevalence of infection among the population, and the two parameters of the KK method decrease generally with a decrease of prevalence in humans. For example, using the two repeated Kato-Katz results as the reference gold standard, the sensitivity of a single stool examination with the three-slide Kato-Katz method was 68.4% - 70.0% in Village A, with an infection rate of 18.6% and 59.6%-69.2% in Village B with a prevalence of 6.6%, respectively [14]. Thus, more than 30% of infected people are misdiagnosed by the Kato-Katz method in endemic regions in China, who are likely to be missed in treatment and continue to serve as infection sources.

**Table 1:** Sensitivity, specificity and predictive value of KK and MHT methods for diagnosing *S. japonicum *infection

Method	Sensitivity (%)	Specificity (%)	*PPV (%)	*NPV (%)	*Prevalence (%)	Reference gold standard	Author and date of publication
*KK	51.1-84.1	100	100	93.2-96.5	13.0-18.4	two repeated KK results (6 slides)	Lin et al. 2008 [13]
	59.6-70.0	100	100	93.3-97.9	6.6-18.6	two repeated KK results (6 slides)	Zhou et al. 2007 [14]
	72.2-100.0	100	100	96.3-100.0	6.2-12.0	combined results of KK(3 slides) and MHT	Xu et al. 2007 [15]
	58.3	100	100	83.5	32.1	MHT	He et al. 2007 [16]
	67.6	100	100	58.9	68.3	seven repeated KK examinations (14 slides)	Yu et al. 2007 [17]
	69.9	100	100	95.63	13.2	combined results of KK(3 slides) and MHT	Zhu et al. 2005 [18]
	50.0	100	100	98.02	3.9	combined results of KK(3 slides) and MHT	Song et al. 2003 [19]
	40-68	100	100	52.5-80.5	36.0-68.3	seven accumulated KK results(14 slides)	Yu et al. 1998 [20]

*MHT	24.0-95.0	100	100	95.2-99.6	6.2-12.0	combined results of KK (3 slides) and MHT	Xu et al.2007 [15]
	79.2	100	100	94.0	23.6	KK(3 slides)	He et al. 2007 [16]
	32.8	100	100	40.9	68.3	seven accumulated KK results (14 slides)	Yu et al. 2007 [17]
	89.8	100	100	98.5	13.2	combined results of KK(3 slides) and MHT	Zhu et al.2005 [18]
	94.4	100	100	99.8	3.9	combined results of KK(3 slides) and MHT	Song et al. 2003 [19]

*PPV = Positive predictive value; *NPV = Negative predictive value; *KK = The Kato-Katz thick smear stool examination; *MHT = Miracidium hatching test;

*Prevalence is calculated based on the reference gold standard

The miracidium hatching test is another traditional approach for assessing *S. japonicum *infection and has been used widely in China for more than five decades [21]. The test is initiated by the concentration of eggs from feces through a nylon tissue bag and suspension in distilled water. Miracidia that hatch from ova are visualized microscopically, and their presence is an indication of infection. The specificity and PPV of the MHT method are high and identical irrespective of the reference gold standards and the infection rates amongst the population, however, the sensitivities of the MHT method range from 24.0% to 95.0%, and the NPV of the MHT method varies from 40.9% to 99.6% (Table 1) [15-19]. The variation range of both the sensitivity and NPV of the MHT method was larger than that of the KK method (Table 1) [13-20]. As shown in Table 1[15-19], the result of the hatching test is unstable, and is highly dependent on the infection prevalence among the population and environmental factors such as temperature and water quality (e.g. pH). For example, when the combined results of seven repeated Kato-Katz examinations and hatching tests were used as the gold standard, the sensitivity of the hatching test was only 32.8% (less than 67.6% for a single Kato-Katz examination) in Zhuxi Village, while in Zhonjiang Village, the hatching test detected more positive cases than the Kato-Katz test did (prevalence 31% vs. 24%) [20]. This method has not been standardized for quantitative measurement, whereas, the KK method can obtain egg counts (EPG, eggs per gramme of feces) which gives an indication of infection intensity (useful information for control programs), more importantly, even under optimal conditions, only 50% to 70% of eggs will hatch, with light infections being missed [17,22].

Ideally multiple stool examinations should be performed in order to reduce the false-negative results; however, repeated stool collection and examination require a lot of time and manpower. Thus, it is impractical to initiate such a strategy at the national level for routine schistosomiasis control programs. As an ideal field method for the detection of schistosome eggs in stool samples is still not available, the use of traditional parasitological tests poses a great challenge in identifying infected cases particularly in low transmission settings.

### Indirect immunological techniques

Indirect immunodiagnostic assays, i.e. detection of schistosome-specific antibodies, have a long history in China. There are many variations of indirect immunological methods [23]. However, Circumoval Precipitin Test (COPT), and Indirect Hemagglutination Assay (IHA) are historically the most widely used immunodiagnostic tests in China, while various forms of Enzyme-linked immunosorbent assay (ELISA) and Dipstick dye immunoassay (DDIA) have become more important over the past 10-20 years [23].

COPT has been widely used in China for almost 50 years [24] and undergone a series of modifications including the application of a variety of labelling techniques in attempts to standardize the testing system including antigen preparation and testing protocol. The COPT has a high sensitivity (94.1%-98.6%) and a low false-positive rate (2.5%-3.6%) in healthy people from a non-endemic area [25]. However, with repeated chemotherapy in the endemic areas, the sensitivity of this method decreased to 72.2% - 85.8% (Table 2) [16,19,26]. The NPV of the COPT method is high (more than 87%), but the PPV of this method is low (from 31.7% to 74.9%) (Table 2) [16,19,26]. In addition, this assay is time-consuming and comparatively complicated and requires microscopy, limiting its wide application in China.

**Table 2:** Sensitivity, specificity and predictive value of COPT and IHA methods for diagnosing *S. japonicum *infection

Method	Sensitivity (%)	Specificity (%)	*PPV (%)	*NPV (%)	*Prevalence (%)	Reference gold standard	Author and date of publication
*COPT	85.8	54.1	36.6	92.5	23.6	KK (3 slides)	He et al. 2007 [16]
	82.8	57.7	48.1	87.7	32.1	MHT	He et al. 2007 [16]
	85.2	95.6	74.9	97.7	13.4	MHT	Zhu et al. 2005 [26]
	72.2	93.7	31.7	98.8	3.9	combined results of KK (3 slides) and MHT	Song et al. 2003 [19]

*IHA	80.0	93.6	18.8	99.6	1.7	MHT	Zhang et al. 2010 [27]
	69.7	89.4	36.8	96.8	8.9	two repeated KK results (12 slides)	Lin et al. 2008 [28]
	97-100	60-77	19-30	100.0	3.1-14.2	KK examination (3 slides)	Zhou et al. 2008 [29]
	83.7-92.3	55.8-67.3	16.6-30.2	93.7-99.2	6.6-18.6	two repeated KK results (6 slides)	Zhou et al. 2007 [14]
	76.0-85.6	35.7-63.6	8.9-22.2	94.8-97.7	6.2-12.0	combined results of KK (3 slides) and MHT	Xu et al. 2007 [15]
	80.3	48.4	77.0	53.3	68.3	seven repeated KK examinations (14 slides)	Yu et al. 2007 [17]
	66.7-100.0	67.6-91.6	2.9-15.8	97.6-100.0	0.3-5.7	two repeated KK results (6 slides)	Wang et al.2006 [30]
	82.1	71.1	26.8	96.9	9.2	KK (3 slides)	Li et al. 2002 [31]

*PPV = Positive predictive value; *NPV = Negative predictive value; *COPT = Circumoval precipitin test; *IHA = Indirect hemagglutination assay;

*Prevalence is calculated based on the reference gold standard

The IHA with soluble extracts of schistosome eggs was developed and applied in late the 1960s, and is secondary only to COPT in its long history of use in China. Currently, the IHA method is still an extensively employed method for community diagnosis and screening of people targeted for chemotherapy, owing to its relatively high sensitivity, simplicity and rapidity in field use compared with the KK and MHT methods. However, with repeated chemotherapy in the endemic areas, the PPV of the IHA method is low, and most reported PPVs are less than 37% (Table 2) [14,15,17,27-31]. The sensitivity of the IHA method varies from 69.7% to 100.0%, and its specificity ranges from 35.7% to 93.6% (Table 2) [14,15,17,27-31]. In addition, the cross-reaction with *Paragonimus westermani *was 64%-84% with soluble egg antigen (SEA) and 31.3% with purified egg antigen [32]. These disturb its continued wide application in China.

ELISA was first described by Engvall and Perlmann [33]. In the late 1970s, the classical ELISA using crude antigens of the parasite emerged for the diagnosis of schistosomiasis, this was regarded as the tool being most likely to meet stringent requirements for field use in terms of reliability, sensitivity, and specificity; in the following years many different variations have been developed for field uses (e.g. Dot-ELISA, SPA-ELISA). Table 3[13-16,19,26,27,30,34] summarizes the sensitivity, specificity, and predictive value of the ELISA method for diagnosing *S. japonicum *infection. The sensitivity of this method varies from 65.5% to 100%, whilst most reported specificities of this method are less than 60% (Table 3) [13-16,19,26,27,30,34]. The NPV of the ELISA method is high ( > 88.0%), but most of the reported PPVs of this method are very low. Although this technique has relatively high sensitivity, an ELISA reader is required to process samples and the delay time (e.g. from sample processing to result reading) is usually 2-3 hours.

**Table 3:** Sensitivity, specificity and predictive value of ELISA and DDIA methods for diagnosing *S. japonicum *infection

Method	Sensitivity (%)	Specificity (%)	*PPV (%)	*NPV (%)	*Prevalence (%)	Reference gold standard	Author and date of publication
*ELISA	80.0	81.6	7.1	99.57	1.7	MHT	Zhang et al. 2010 [27]
	79.3-87.4	38.9-53.5	20.8-24.6	93.1-94.4	13.3-18.5	two repeated KK results (6 slides)	Lin et al.2008 [13]
	88.4-96.2	38.4	9.9-24.7	93.5-99.3	6.6-18.6	two repeated KK results (6 slides)	Zhou et al. 2007 [14]
	95.0	20.4	26.9	93.0	23.6	KK (3 slides)	He et al.2007 [16]
	98.2	23.8	37.9	96.6	32.1	MHT	He et al. 2007 [16]
	65.8	51.7	8.3-15.7	91.7-95.8	6.2-12.0	combined results of KK (3 slides) and MHT	Xu et al.2007 [15]
	65.5	59.4	12.7	95.0	8.3	combined results of three repeated KK (12 slides) and MHT	Chen et al.2007 [34]
	88.9-100.0	43.2-92.2	3.1-18.6	99.2-100.0	0.3-5.7	two repeated KK results (6 slides)	Wang et al.2006 [30]
	92.1	90.4	59.6	98.7	13.4	MHT	Zhu et al.2005 [26]
	83.3	84.3	77.2	88.8	3.9	combined results of KK (3 slides) and MHT	Song et al.2003 [19]

*DDIA	75.0-95.5	37.2-78.7	0.5-18.4	98.6-99.9	0.4-8.2	combined results of KK (3 slides) and MHT	Xu et et al. 2011 [38]
	80.0	92.2	15.5	99.6	1.7	MHT	Zhang et al. 2010 [27]
	75.3	55.1	10.0-18.6	94.2-97.1	6.2-12.0	combined results of KK (3 slides) and MHT	Xu et al.2007 [15]
	95.1	37.4	41.8	94.2	32.1	MHT	He et al.,2007 [16]
	92.5	33.0	29.9	93.4	23.6	KK (3 slides)	He et al.2007 [16]
	44.8	69.8	87.6	21.0	8.3	combined results of three repeated KK (12 slides) and MHT	Chen et al. 2007 [34]
	96.6	96.3	80.2	99.5	13.4	MHT	Zhu et al.2005 [26]
	94.4	87.4	23.3	99.7	3.9	combined results of KK (3 slides) and MHT	Song et al.2003 [19]
	94.9	40.8	17.1	98.4	9.2	KK (3 slides)	Li et al. 2002 [31]

*PPV = Positive predictive value; *NPV = Negative predictive value; * ELISA = Enzyme-linked immunosorbent assay; *DDIA = Dipstick dye immunoassay; *Prevalence is calculated based on the reference gold standard

A meta-analysis [35] showed that the sensitivity ranged from 40.7% to 95.1% for IHA, from 23.3% to 97.3% for ELISA; the false positive rate was 11.0% to 55.6% of IHA and 16.3% to 79.6% of ELISA, and the results of the meta-analysis indicated that IHA had more preferable diagnostic properties than the ELISA.

The DDIA, a new immunodiagnostic assay, has been developed in recent years [36,37]. This method is basically a chromatography technique using soluble egg antigen (SEA) of *S. japonicum*, labeled with a dye as the indicator system. Table 3[15,16,19,26,27,31,34,38] shows that the sensitivity and NPV of the DDIA method are high (≥75% for sensitivity, > 94% for NPV) except for one report [34], but the reference gold standard of this report is closer to the 'true' than that of other reports. The specificity and PPV of this method show very large discrepancies, e.g. the specificity is from 33.0% to 96.3% and PPV ranges from 0.5% to 87.6% (Table 3) [15,16,19,26,27,31,34,38]. In addition, the DDLA has a high cross-reaction with paragonimiasis (70.0%) [36].

Although most of the reported sensitivities and NPV of these antibody detection assays were relatively high, the discrepancy in both the reported specificities and reported PPV of these methods was very large, and moreover, most of these reported specificities and PPV were very low (Table 2, Table 3) [13-17,19,26-31,34,38]. This discrepancy may be attributed to the following factors [35]: (a) the choice of the reference gold standard. Examination for parasite eggs in excreta are often used as the gold standard when evaluating the performance of immunodiagnostic tests for schistosome infections [12], of the parasitological examinations, KK and MHT methods, or combined results of KK and MHT are often used as gold standards when testing immunological techniques in China, particularly in population-based studies, the number of the Kato-Katz thick smears examined ranged from 3 slides to 14 slides (Table 2, Table 3) [13-17,19,26-31,34,38]. More 'true' infections were observed in an endemic area with an increase of the number of KK thick smears being examined due to the low sensitivity of the KK method [13]. So, the difference in both reported specificities and reported PPV of an immunodiagnostic method were large when the different tests (KK, MHT or combined results of KK and MHT) were used as a gold standard. Hence, an appropriate gold standard for testing field-applied immunological techniques would be the use of multiple stool examinations, for example, repeated KK examinations or the KK method combined with the MHT method; (b) infection prevalence and intensity in a community; (c) different diagnostic agents of schistosomiasis; and (d) size of the sample. The low specificity is likely to result in a high 'false-seropositivity', i.e. the high proportion of seropositive individuals were classified into negative groups of stool examination in endemic regions, especially in the areas with relatively high endemicity, where re-infection and repeated chemotherapy are very common. For example, the false-positive rate of IHA was 44.2% in a village with a prevalence of 18.6%, and 32.7% in another village with a prevalence of 6.6%, while that of ELISA assays was higher (61.6%), when the two accumulated Katz-Kato results were designated as the gold standard [14]. Apart from the low specificity and PPV, these assays have generally a high cross-reaction with other parasites e.g. *P. westermani *[22]. Among all obstacles of antibody-based immunodiagnostic tests, the major one is that these currently available assays cannot distinguish active infection from previous infection or re-infection, which results in a high false-positive, thereby causing difficulties in identifying infected individuals for selective chemotherapy, and assessing the effectiveness of intervention.

### Direct immunological tests

Previously, schistosome-derived antigens, through immunoassays, were shown to be present in the circulation and/or excreta of infected hosts [39], prompting considerable research on their potential for immunodiagnosis of schistosomiasis. In the early 1980s, research was initiated first by Qian and Deelder [40] to explore detection of the circulating anodic antigen (CAA) for immunodiagnosis of *S. japonicum *infection in China. The results demonstrated that the detection limit was from 10 to 0.5-0.25 ng/ml by use of monoclonal antibody (mAb)-defined CAA series. Thus far, a lot of testing systems based on monoclonal or polyclonal antibodies for detection of different target antigens had been developed in more than 10 laboratories in China.

In 1993, a collaborative study focusing on evaluation testing systems for antigen detection was conducted in China [41]. The results showed that most tests involved in the detection of different circulating antigens were not satisfactory, showing high false seroreactivity ranging from 24 to 46% and low sensitivity ranging from 15 to 73% in chronic and light infections [41]. Afterwards a special national program was carried out. This was aimed at improving the diagnostic capacity of existing assays and also seeking novel probes with emphasis on those that could be promising for the use of monitoring the efficacy of chemotherapy. This was initiated under the supervision of the Schistosomiasis Expert Advisory Committee, Ministry of Health. Then in 1995, 14 testing systems, of which 13 for antigen detection and 1 for antibody detection, from 12 laboratories were brought to Wuhan city for a collaborative evaluation [42]. Among the 13 assays for antigen detection, 9 showed high specificity with above 90% but only 3 of the assays gave above 68% sensitivity, of which the highest was 81%, in chronic and light infections. Furthermore, there was also no clear-cut evidence that antigen-based assays could provide useful correlation with levels of infection intensity [41].

## Conclusion

### Pressing need for improved diagnostics

After five decades' of effort, China has made great achievements in schistosomiasis control and the infection prevalence and intensity of schistosomiasis in humans have decreased to a relatively low level [6,8]. The achievement, to a large extent, is attributable to praziquantel-based chemotherapy, and prompts an increasing understanding of the need for sufficiently sensitive and specific diagnostic or screening techniques of schistosomiasis in low transmission settings. However, we are currently in a diagnostic dilemma for *S. japonica *- the direct parasitological techniques have become relatively insensitive due to widespread chemotherapy that results in generally low worm burdens, which leads to less efficiency in low transmission settings and in post-treatment situations [14,17,20]. Other diagnostic alternatives include detection of antibodies or circulating antigens in serum. Antibody detection assays with relatively high sensitivity but generally low specificity, do not differentiate between current and cured infections, which results in the difficulties in determining prevalence, identifying true infected individuals for selective chemotherapy and assessing the effectiveness of intervention including follow-up of chemotherapy. The detection of circulating antigens is a highly specific assay, but has not been shown to be more sensitive than the detection of eggs in areas of low endemicity [42-45]. Therefore, it is very difficult to select the most appropriate diagnostic detection methods for *S japonicum *in the areas where re-infection and repeated chemotherapy are frequent [14]. On the one hand, if every person with a positive result from antibody-based immunodiagnostic assays is treated with praziquantel year after year, a considerable number of previous infections will be treated repeatedly, which results in inappropriate use of praziquantel and reduction in chemotherapy compliance [14]. On the other hand, if these immunodiagnostic assays are only used for preliminary screening and all those with positive results of immunodiagnostic assays are subject to stool examination to confirm infection, then only the individuals with positive egg counts are treated, a higher proportion of infections may be missed by this method than by using only the fecal examination [14]. It is suggested that the insensitivity or non-specificity of currently applied diagnostic methods conspire to produce inaccurate estimates of disease impact, and this threatens the successful drive towards full control of schistosomiasis [46]. Hence, the search for a robust diagnostic test that can be applied in field situations is essential and should be given high priority.

Recently, polymerase chain reaction (PCR) based assays have been developed to detect *S. mansoni*, *S. haematobium *and *S. japonicum *infections, and have shown potential as a highly sensitive and specific technique for detection of parasite DNA in feces or sera and plasma, especially in regions with low intensity infections [47-54].

## Competing interests

The authors declare that they have no competing interests.

## Authors' contributions

YBZ and QWJ wrote the manuscript together, YBZ and HMZ summarized the Tables, and all have read and approved this final version.
